# Evaluation of the effects of extremity elongation on brachial plexus nerves via intraoperative neuromonitoring in patients undergoing reverse total shoulder arthroplasty

**DOI:** 10.1186/s12891-026-09633-z

**Published:** 2026-02-19

**Authors:** Dağhan Koyuncu, Alper Şükrü Kendirci, Ali Erşen

**Affiliations:** İstanbul Medical School, Department of Orthopedics and Traumatology, İstanbul University, Millet Cad. No: 118 34093 Capa Fatih, İstanbul, Turkey

**Keywords:** shoulder prosthesis, intraoperative neurophysiological monitoring, brachial plexus, peripheral nerve injuries, limb lengthening

## Abstract

**Background:**

Reverse total shoulder arthroplasty (RTSA) is one of the most common treatment method for irreparable rotator cuff tear. One of the significant complications of this surgery is nerve injury. This study aimed to identify the patterns of brachial plexus nerve involvement and to determine the surgical stages associated with increased nerve vulnerability during reverse total shoulder arthroplasty using intraoperative neuromonitoring (IONM). Additionally, the relationship between extremity lengthening, acromiohumeral distance (AHD) changes and intraoperative nerve events were evaluated.

**Methods:**

Twenty patients diagnosed with rotator cuff tear arthropathy who underwent total reverse shoulder arthroplasty in a single center were included in the study. Demographic data, preoperative shoulder range of motion (ROM), and functional scores were recorded. Radiological measurements were performed to evaluate extremity lengthening and preoperative acromiohumeral distances (AHD) differences on both shoulders. The relationship between these values and intraoperative motor-evoked potential (MEP) amplitude drops was analyzed. The nerves affected and the corresponding surgical stages were documented. Statistical analysis was performed to assess the relationship between nerve involvement and preoperative variables.

**Results:**

Among the 20 patients evaluated, the musculocutaneous and radial nerves were most frequently affected, particularly during the glenoid preparation stage. Smoking history (*p* = 0.019) and increased preoperative internal rotation (*p* = 0.05) were significantly associated with higher risk of nerve involvement. Binomial logistic regression analysis shows that smoking history is independently associated with a higher risk of intraoperative nerve events (OR 11.49, 95% CI 1.03-128.57; *p* = 0.048). A preoperative difference of less than 3.7 mm in preoperative AHD between the operated and contralateral sides reduced the risk of nerve involvement by 4.2 times.

**Conclusions:**

The musculocutaneous and radial nerves were found to be most commonly affected, during glenoid preparation phase, due to the excessive traction and improper positioning of the retractors. Lower preoperative AHD difference of less than 3.7 mm increases the risk of nerve events. The use of IONM during RTSA surgery has a preventive effect along with its diagnostic use.

**Level of Evidence:**

4, prospective case series.

**Supplementary Information:**

The online version contains supplementary material available at 10.1186/s12891-026-09633-z.

## Background

Rotator cuff tears are common in the elderly population, with up to 40% of individuals over 70 years of age affected [1, 2]. Cuff tear arthropathy (CTA) can occur if rotator cuff tears are not diagnosed and treated appropriately. Due to the loss of dynamic and static shoulder joint stabilizers, shoulder stability and biomechanics are affected [3, 4]. In individuals with untreated rotator cuff tears, the disruption of shoulder force couples may lead to superior migration of the humeral head, ultimately resulting in glenohumeral articular cartilage damage due to a shift in the center of shoulder rotation [5, 6].

Reverse total shoulder arthroplasty (RTSA) is a non-biological joint replacement method which was first described by P. Grammont in 1982 [7]. It involves replacing the native concave glenoid with a convex glenosphere, and the convex humeral head with a concave humeral tray. The primary goal of RTSA is to compensate for damaged rotator cuff tendons with deltoid for shoulder range of motion [8]. Medialization and distalization of the shoulder’s center of rotation are key design principles aimed at recruiting more deltoid fibers to improve joint stability and mobility [9, 10].

Nerve injury with clinical findings after primary RTSA is reported to range from 0.9% to 6% [11, 12]. During RTSA surgery, such injuries can occur due to retractor placement or traction effect caused by distalization of the center of rotation and extremity lengthening [8, 13]. This traction effect can cause epineuroclasis and endoneuroclasis. Recent research illustrates that intraoperative loss of nerve conduction has a poor prognosis for nerve stretch injuries [14, 15]. Intraoperative neuromonitoring (IONM) may help detect and prevent these events. IONM assesses the sensory dorsal column – medial lemniscus pathway using somatosensorial evoked potentials (SSEP) and motor pathways using motor evoked potentials (MEP). The use of IONM for shoulder surgery is still limited in the literature, with available studies focusing on Latarjet procedure [16] and shoulder arthroplasty [17, 18]. However, the literature remains inconclusive regarding which nerves are the most vulnerable and which surgical stages carry the highest risk.

The aims of this study are: (i) to identify which brachial plexus nerves are at the highest risk during RTSA; to determine which stage of RTSA surgery is associated with increased nerve involvement; (ii) to evaluate the association between preoperative patient specific factors such as extremity length, AHD, and nerve involvement. We hypothesized that intraoperative nerve involvement during RTSA is not uniformly distributed among brachial plexus nerves or surgical stages and preoperative AHD difference and extremity lengthening are associated with the occurrence of intraoperative nerve events which can be detected by IONM.

## Material and methods

### Study design

This study is a prospective case-series conducted between January-November 2024 in a tertiary clinic by a single experienced shoulder surgeon (A.E.). Patients with the diagnosis of cuff tear arthropathy diagnosis who were treated with reverse total shoulder arthroplasty were included. All nerve involvements (without exclusion according to the cut-off value) were recorded and analyzed in order to explore the primary aim of this study, which is to identify the nerve at the highest risk and the stage associated with increased nerve involvement. Patients were divided into two groups according to the presence of > 30% nerve involvement. Intergroup comparison of demographic data, functional scores, range of motion and radiological studies were performed.

### Patient selection

Inclusion criteria for this study were: (1) patients over 55 years; (2) diagnosis of CTA with Hamada stage 3 or higher; (3) treatment with lateralized onlay RTSA; (4) voluntary participation with informed consent.

Exclusion criteria for this study were: (1) history of proximal humerus fracture; (2) revision RTSA; (3) previous or active shoulder infection; (4) preoperative upper extremity sensory or motor nerve deficit; (5) need for additional procedures due to intraoperative complications.

All patients involved in this study were thoroughly informed about the study and written informed consent was obtained consecutively.

### Clinical evaluation

Patients presenting to the orthopedics and traumatology outpatient clinic with shoulder pain were evaluated. Those with CTA Hamada stage 3 or higher were selected. Demographic and clinical data including age, sex, height, weight, body mass index (BMI), dominant side, comorbidities, medications, surgery history, and smoking history were recorded.

Active and passive ranges of motion of both upper extremities were evaluated. Upper extremity sensory and motor neurological examinations were performed.

Patient-reported outcome measures including visual analogue scale (VAS), Constant-Murley scores [19] and American Shoulder and Elbow Society (ASES) scores [20] were assessed and documented.

### Radiological evaluation

Preoperatively, anteroposterior (AP) and true AP x-rays of both humeri were obtained. Magnetic resonance images (MRI) were used to assess the extent of rotator cuff tear, fatty infiltration and articular cartilage and subchondral bone changes. CTA was confirmed radiographically.

**Fig. 1:**
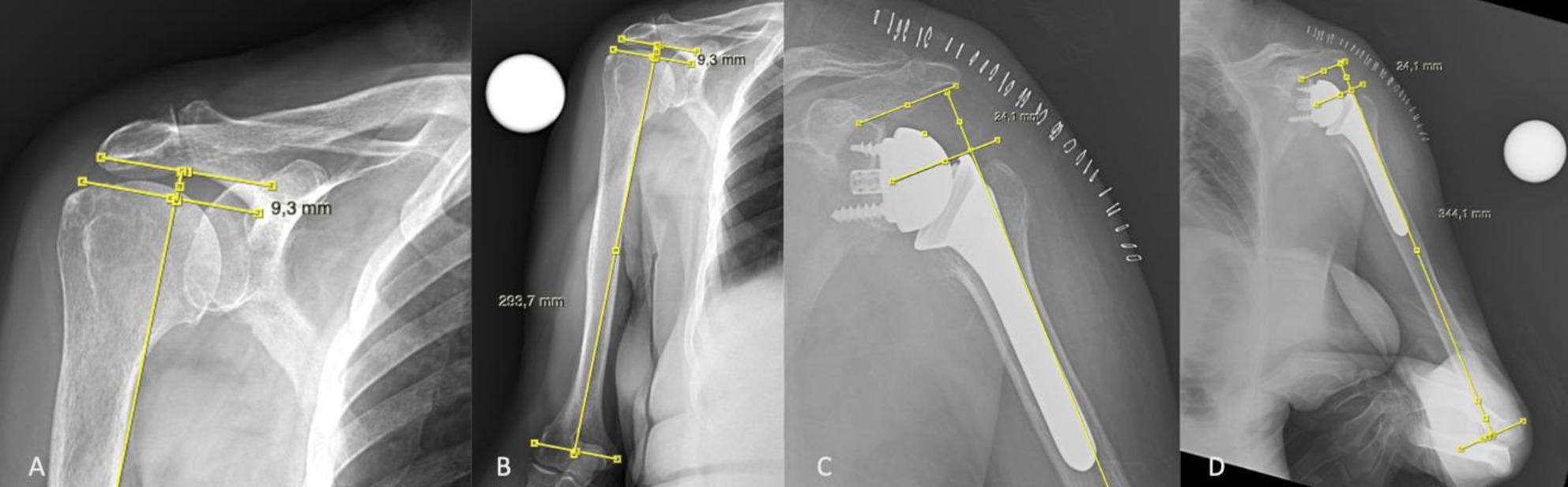
**A**, preoperative extremity length measurement on AP x-ray; **B**, preoperative acromiohumeral distance measurement on AP x-ray; **C**, postoperative extremity length measurement on AP x-ray; 1D, postoperative acromiohumeral distance measurement on AP x-ray

Humeral length and acromiohumeral distances were measured on both sides using a standardized true AP view according to Ladermann et al. [12]. The transepicondylar axis was marked, and a parallel line was drawn immediately superior to the most superior contour of the humeral head. The vertical distance between these lines was recorded as humeral length (Fig. 1A). Acromiohumeral distance was measured from the inferolateral edge of acromion to the superior humeral head contour (Fig. 1B). Postoperative measurements were obtained for the operated side (Fig. 1C, D). The measurements were performed by a single orthopedic surgeon.

### Surgical preparation

All patients received general anesthesia. Neuromuscular blocking agents were administered during endotracheal intubation. During the procedure, neither inhalational anesthetics such as nitrous oxide nor additional neuromuscular blocking agents were administered, as these agents may interfere with MEP recordings. Total intravenous anesthesia (TIVA) was preferred. No interscalene nerve block was administered to any patient in order to eliminate brachial plexus depression as a confounder.

After intubation, patients were positioned in the beach-chair position. While positioning, the posterior aspect of the scapula was left unsupported.

**Fig. 2:**
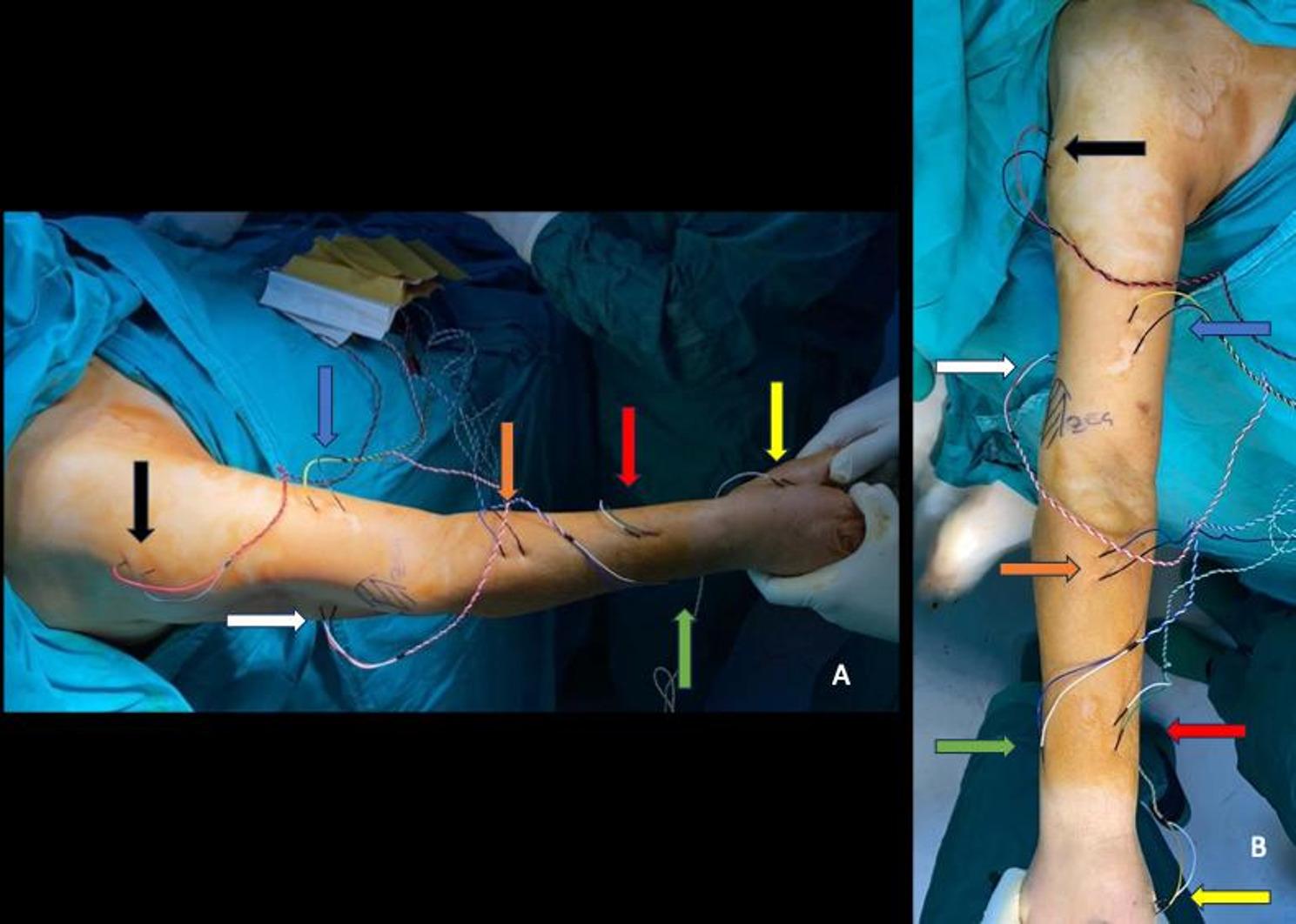
IONM electrode placement. **A** Electrode placement from lateral. **B** Electrode placement from anterior. Black, deltoid m.; white, triceps brachii m. ; blue, biceps brachii m., orange, brachioradialis m.; red, extensor carpi radialis longus m.; green, abductor pollicis brevis m.; yellow, 1^st^ dorsal interosseous m

After the patient was positioned and draped, sterile IONM electrodes were inserted. Both SSEP and MEP monitoring were performed throughout the procedure. For SSEP monitoring, median nerve was stimulated. For MEP evaluation, electrodes were placed in the deltoid, biceps brachii, extensor carpi radialis longus, triceps brachii, abductor pollicis brevis, abductor digiti minimi and cranium as described in a previous study done by Shinagawa et al. (Fig. 2) [21]. A ≥ 30% decrease in MEP amplitude was considered a meaningful decrease in amplitude [22].

The operation was divided into seven stages: incision, dislocation, humeral preparation, glenoid preparation, glenosphere placing, reduction, and skin closure. Evoked potentials were recorded at the beginning of each stage.

All amplitude decreases for SSEP and MEP were recorded. The surgeon was notified as soon as amplitude decreases were seen. In case of a signal decrease, all retractors were removed and evoked potentials were measured. If the amplitude reduction persisted, the extremity was returned to a neutral position and measurements were repeated. For all patients, amplitude changes in every surgical stage were investigated, the highest amplitude change was accepted as the main nerve event.

### Surgical procedure

**Fig. 3:**
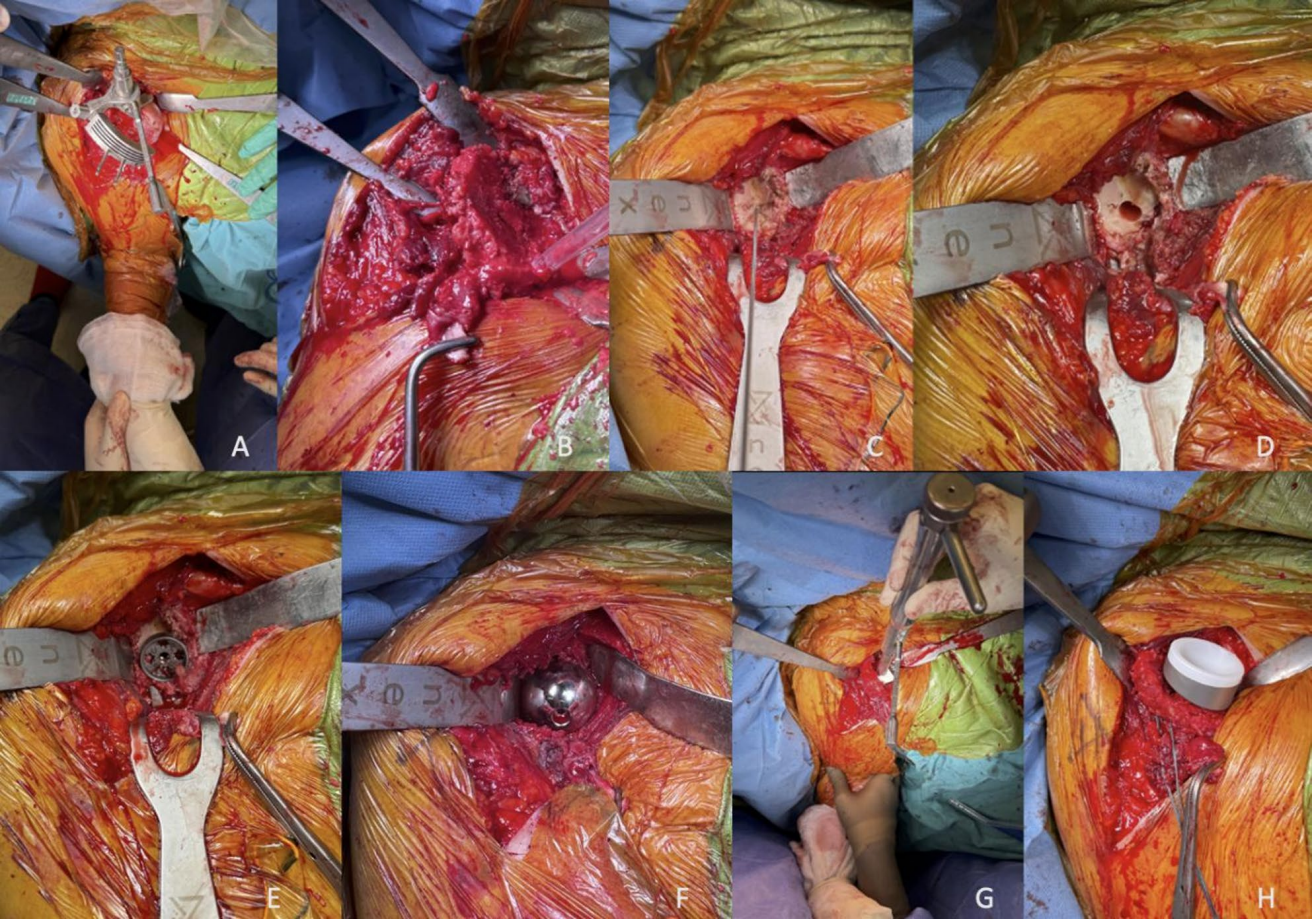
Surgical procedure. **A**, humeral cutting guide, in 20˚ retroversion; **B**, humerus after anatomical neck osteotomy; **C**, glenoid guide wire position; **D**, peg hole after reaming; **E**, glenoid base plate positioning after reaming; **F**, glenosphere positioning; **G**, humeral stem insertion in 20˚ retroversion position; **H**, humeral component and humeral tray before reduction

All surgeries were performed by a single shoulder surgeon. RTSA with lateralized humeral stems was used for all patients. After standard deltopectoral approach, cephalic vein was retracted laterally. The clavipectoral fascia was excised and the retractors were positioned over the conjoint tendon. The long head of biceps brachii tendon was identified and tenotomized. The subscapularis tendon was tenotomized from the lesser tuberculum and the rotator interval was released. Then, the humeral head was dislocated, and an intramedullary guide was inserted. Reaming was performed until the appropriate size was reached. The cutting guide was then placed and the humeral head was resected at the bone – cartilage junction. Glenoid preparation began with the release of MHGL and anterior IGHL. Then, posterior, anterior and inferior retractors were positioned. A guidewire was placed in the center of the glenoid with a 10˚ cephalad tilt. The cartilage surface was reamed and the peg hole was prepared. Glenoid base plate was inserted and secured with two 4.5 mm locking screws: one directed toward the coracoid base and the other toward scapular pillar. Glenosphere was placed and secured with a central screw. Then, humeral stem was inserted. Appropriate size humeral tray was tested and inserted. Following reduction, biceps brachii tenodesis was performed onto conjoint tendon. Subscapularis tendon could not be repaired due to the use of lateralized humeral component. A drain was inserted and the incision was closed (Fig. 3).

### Post-operative rehabilitation

Postoperatively, all patients were evaluated for neurological function. All patients were instructed to use a sling for four weeks. Elbow range of motion exercises and wound care were initiated on postoperative day one. All patients followed a standardized shoulder arthroplasty physiotherapy protocol. Sutures were removed at the second week. Patients were monitored biweekly, and neurological assessments were performed at each visit. Postoperative electromyography (EMG) was not performed since none of the patients demonstrated any sensory or motor deficit at follow-up.

### Study size

Sample size estimation was based on our primary outcome, intraoperative nerve involvement demonstrated by MEP changes. The calculation was performed considering the occurrence of nerve events as a binary outcome, using the effect size assumptions derived from a previously published IONM study with a similar design. A sample size of 16 patients was calculated to be sufficient to achieve statistically significant results, assuming an effect size of 0.6 and a margin of error of 0.05 [23].

### Statistical analysis

Data were analyzed using NCSS (Number Cruncher Statistical System) 2020 Statistical Software (NCSS LLC, Kaysville, Utah, USA). Shapiro-Wilk test and box plot graphs were used to assess the normal distribution of the data. For non-normally distributed data (age, height, functional scores, ROM), Mann-Whitney U test was used for intergroup analysis of radiological data comparison, and the Wilcoxon signed rank test was used for intragroup analysis of AHD difference. Categorical variables such as the history of DM, RA and smoking were compared using Fisher’s exact test and Fisher-Freeman-Halton test. A 95% confidence interval was used, and *p* < 0.05 was considered statistically significant. ROC curve analysis was performed to evaluate the predictive value of the preoperative acromiohumeral distance difference between the operated and contralateral shoulders for intraoperative nerve events. The optimal cut-off was determined based on the maximum Youden index. Binomial logistic regression analysis was performed to assess the association between smoking, preoperative internal rotation degrees and extremity lengthening with intraoperative nerve events.

While a ± 30% amplitude decrease threshold in MEP was used to evaluate the relationship between demographic and radiological variables and nerve involvement, this threshold was not applied when analyzing surgical stages and nerve-specific involvement due to the low number of threshold-positive events in these subgroups, which limited statistical interpretability.

## Results

A total of 22 patients who underwent surgery between January and November 2024 were initially enrolled in the study. One patient was excluded after conversion to hemiarthroplasty due to intraoperative glenoid fracture. Another patient was excluded due to intraoperative hemodynamic instability, which prevented the surgery from proceeding. Therefore, 20 patients were included in the final analysis.

Fourteen patients were female. The median age was 70.5 (55–77) years. 15 patients (75%) were operated on the right shoulder. Seven patients (35%) had a smoking history. The mean surgical duration was 94.75 ± 6.26 min.

None of the patients experienced a significant decrease in SSEP amplitude. However, 8 patients (40%) showed a significant (> 30%) decrease in MEP amplitudes. A total of 76 nerve alerts with a median decrease of 9.5% were recorded, regardless of the threshold value (0–66).

### Evaluation of the nerve events and surgical stages

Overall, 18 patients had at least one nerve event. 15 patients had musculocutaneous nerve, 15 patients had radial nerve, 14 patients had posterior interosseous nerve, 13 patients had median nerve, 10 patients had ulnar nerve and 9 patients had axillary nerve involvement (Table 1).

**Table 1:** Number of nerve events for each nerve and number of nerve events for each phase

Nerves	*N*	%	Surgery Phases	*N*	%
Musculocutaenous *n*.	15	19.7	Dislocation	15	19.7
Radial n.	15	19.7	Glenoid preparation	17	22.4
Posterior Interosseous n.	14	18.4	Glenosphere placement	12	15.8
Median n.	13	17.1	Humeral preparation	15	19.7
Ulnar n.	10	13.2	Reduction	13	17.1
Axillary n.	9	11.8	Skin closure	4	5.3

When MEP alerts were compared across different surgical stages, the glenoid preparation stage had the highest number of nerve alerts (*n* = 17). During dislocation, 15 alerts, during glenosphere placing, 12, during humeral preparation, 15, during reduction 13 and during skin closure, 4 alerts were recorded. All nerve alerts returned to baseline after retractors were taken out and the arm was positioned in a neutral position (Table 1).

### Evaluation of radiologic findings

**Fig. 4:**
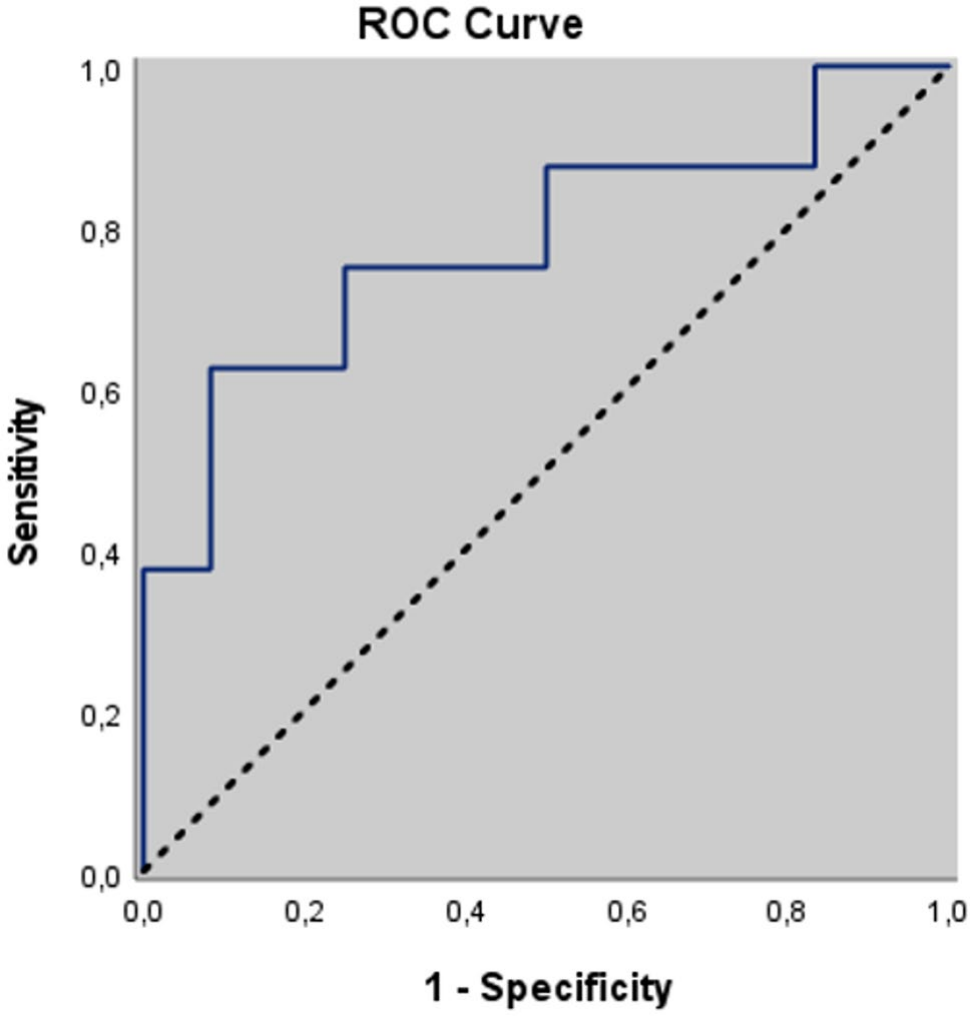
ROC curve for the relationship between preoperative acromiohumeral distance difference and nerve events, area under curve is 78.1%

The difference in preoperative bilateral extremity lengths (*p* = 0.05) and preoperative bilateral acromiohumeral distances (*p* = 0.003) was significantly lower for the operated side. The median increase in extremity length between preoperative and postoperative measurements was 2.4 (-0.9-6.6) cm. In binomial logistic regression analysis, extremity lengthening was not associated with nerve events (*p* = 0.53). The difference between preoperative bilateral acromiohumeral distances was significantly higher in patients who experienced nerve events (*p* = 0.05). For greater than 3.7 mm cutoff value of preoperative bilateral acromiohumeral distance difference, the risk of nerve involvement was increased by 4.2 times with 75% sensitivity and 50% specificity. The area under the ROC curve (AUC) was 78.1% (Table 2) (Fig. 4).

**Table 2:** Cutoff value and ROC curve analysis for preoperative acromiohumeral distance difference

	ROC Curve									*p*
	Cutoff value (mm)	Senstivity	Specificity	Positive predictive value	Negative predictive value	Area under curve		%95 CI		
Preoperative AHM distance difference (cm)	***≥3,7***	75.0	50.0	50.0	75.0	0.78		0,558-1,000		*0.04*

### Other clinical features

A history of smoking was found to significantly increase the number of intraoperative nerve events (*p* = 0.02). There was no significant relationship between nerve events and history of previous surgery, rheumatoid arthritis, diabetes mellitus or BMI (Table 3). Binomial logistic regression analysis showed that smoking history is independently associated with a higher risk of intraoperative nerve events (OR 11.49, 95% CI 1.03-128.57; *p* = 0.048) (Table 4).

**Table 3:** Demographic data, previous surgery history, comorbidities and smoking history of patients with and without intraoperative nerve event. Smoking history is significantly associated with increased number of nerve events

		Intraoperative Nerve Events		*p*
		(-) (*n*=12)	(+) (*n*=8)	
Age	*Mean ± SD*	69.6 ± 5,7	66.9 ± 7,1	0.47
Extremity	Right	8 (66.7%)	7 (87.5%)	0.60
	Left	4 (33.3%)	1 (12.5%)	
Previous RC repair	**(-)**	10 (83.3%)	5 (62.5%)	0.35
	(+)	2 (16.7%)	3 (37.5%)	
Diabetes Mellitus	**(-)**	8 (66.7%)	4 (50%)	0.65
	(+)	4 (33.3%)	4 (50%)	
Rheumatoid arthritis	**(-)**	10 (83.3%)	7 (87.5%)	1.00
	(+)	2 (16.7%)	1 (12.5%)	
Height (cm)	*Mean ± SD*	161.8 ± 6.9	159.4 ± 5.3	0.57
Weight (kg)	*Mean ± SD*	77.8 ± 10.1	79.9 ± 7.3	0.62
BMI (kg/m^2^)	*Mean ± SD*	29.7 ± 3.8	31.4 ± 4.1	
Smoking	**(-)**	10 (83.3%)	2 (25%)	0.02
	(+)	2 (16.7%)	6 (75%)	

**Table 4:** Model estimated using binomial logistic regression (*N*= 20). Smoking history is independently associated with a higher risk of intraoperative nerve events

	Binomial Logistic Regression		*p*
	Odds Ratio	%95 CI	
Preoperative internal rotation (per degree	1.07	0.99-1.17	*0*.*084*
Smoking history (yes/no)	11.49	1.03 – 128.57	*0.048*

Higher degrees of preoperative shoulder internal rotation were significantly associated with an increased number of intraoperative nerve events (*p* = 0.05). According to the binomial logistic regression analysis, greater preoperative internal rotation showed a non-significant trend toward increased risk (OR 1.07 per degree, 95% CI 0.99–1.17; *p* = 0.084). No significant relationship was found between other range of motion parameters or functional scores and the incidence of nerve involvement (Table 5).

**Table 5:** Preoperative ROM and functional scores of patients with and without intraoperative nerve event. Higher degrees of preoperative shoulder internal rotation were significantly associated with an increased number of intraoperative nerve events

		Intraoperative Nerve Events		*p*
		(-) (*n*=12)	(+) (*n*=8)	
Flexion (°)	*Mean ± SD*	94.2 ± 31.5˚	70.0 ± 36.3˚	*0.35*
Extension (°)	*Mean ± SD*	15.0 ± 11.7˚	11.3 ± 4.4˚	*0.52*
Abduction (°)	*Mean ± SD*	90.4 ± 36.9˚	78.8 ± 40.5˚	*0.91*
Adduction (°)	*Mean ± SD*	17.9 ± 6.2˚	18.1 ± 6.5˚	*0.97*
Internal rotation (°)	*Mean ± SD*	32.9 ± 14.5˚	46.2 ± 16.0˚	*0.05*
External rotation (°)	*Mean ± SD*	30.4 ± 17.1˚	30.6 ± 15.7˚	*0.73*
Constant score	*Mean ± SD*	53.3 ± 14.1	44.9 ± 11.0	*0.18*
ASES score	*Mean ± SD*	46.8 ± 10.0	40.9 ± 6.2	*0.18*
VAS score	*Mean ± SD*	5.6 ± 1.34	5.9 ± 1.3	*0.73*

## Discussion

Our findings indicate that the musculocutaneous and radial nerves were more frequently affected than other nerves, the glenoid preparation phase was associated with the highest incidence of nerve events, and a preoperative bilateral acromiohumeral distance difference greater than 3.7 mm increased the risk of nerve events by 4.2 times.

Nerve injury is one of the most common complications of shoulder surgeries. Reported incidence rates range from 1 to 4% in total shoulder arthroplasty (TSA) and 2–8% in RTSA [11]. Ladermann et al. reported that among 19 patients included in their study, 9 of the patients had 12 neurological complications which were confirmed by postoperative EMG [24]. In studies utilizing IONM, at least one nerve event was detected in 76.5% of patients undergoing the Latarjet procedure [16], 24% in TSA patients [25] and in 56.7% of RTSA cases [17]. In our series, 8 of 20 patients (40%) demonstrated at least one nerve event. Several factors may be responsible for lower rates of nerve events in our study, including the use of different MEP amplitude decrease cutoff values or the uniform surgical technique performed by a single surgeon.

Specific nerves appear to be more susceptible during RTSA. Malik et al. identified the median nerve as the most frequently affected [26]. In Nagda et al.’s study, 20% of nerve events occurred in the musculocutaneous nerve, and 16.7% occurred in the axillary nerve [17]. The axillary nerve (33.9%) was most commonly affected in TSA and radial nerve (22.6%) was most likely to be affected in RTSA in the Parisien et al. study [18]. In this study, the musculocutaneous and the radial nerves were more frequently affected than the rest of the nerves. Hyperextension and traction during dislocation and humeral preparation phases may account for these findings. Nerve event thresholds vary across different studies. This is another reason for the heterogeneity regarding the most vulnerable nerves during RTSA.

Despite relatively few axillary nerve alerts in our series, its injury is frequently mentioned in the literature. Its proximity to infraglenoid tubercle makes it vulnerable during retractor placement. The axillary nerve is found to be approximately 12 mm near from infraglenoid tubercle in radiological and cadaveric studies [27, 28]. For this reason, electrocautery was avoided during inferior glenoid preparation, and extreme caution was applied during retractor placement in order to minimize axillary nerve injury.

Shinagawa et al. observed significantly more nerve events during glenoid and humeral preparation phases [21]. Similarly, a systematic review by Xiao et al. concluded that these stages posed a greater risk than reduction, which had been considered the most dangerous [29]. Our results are in line with the previous studies as we found that glenoid preparation was associated with the highest number of nerve events. Excessive retraction, aggressive positioning of the extremity, careless soft tissue handling and traction on the brachial plexus during preparation are likely contributors to these events [29].

No postoperative clinical neurological deficits were observed in any of the patients with intraoperative IONM changes. These amplitude decreases, which may be caused by physiological stress rather than an overt nerve injury, suggested the need for surgical adjustments. Although intraoperative MEP decreases are not correlated with postoperative nerve damage, they alerted the surgical team and enabled preventive interventions that may help prevent progression to permanent nerve injury. This underlines the possible preventive utility of IONM rather than a purely diagnostic one.

In Grammont type RTSA, distalization and medialization of the joint center of rotation are essential for joint stability and deltoid activation. However, excessive distalization places the brachial plexus under tension. Ladermann et al. defined a cutoff value for distalization and extremity elongation. They concluded that more than 2 cm of extremity elongation is linked to higher rates of clinical neurological complications [24]. In this study, median extremity elongation was found to be 2.4 (-0.9-6.6) cm and extremity elongation was not associated with intraoperative nerve events in logistic regression analysis. This finding suggests that extremity elongation alone may not predict intraoperative nerve event occurrence. Nevertheless, future studies with larger cohorts could define a threshold value for extremity elongation.

This study introduces a novel finding regarding preoperative acromiohumeral distance difference. ROC analysis demonstrated 4.2-fold lower risk of nerve events in patients with an acromiohumeral difference of less than 3.7 mm. To our knowledge, this is the first study to propose a cutoff value for acromiohumeral distance difference in predicting intraoperative nerve events. As CTA progresses, unopposed deltoid force vector pulls the humeral head superiorly. In later stages of CTA, acromiohumeral distance decreases, soft tissue contractures occur; thus, during RTSA surgery, excessive traction may be required in order to achieve distalized center of rotation. Excessive traction may result in detrimental effects on peripheral nerves. Preoperative planning should consider this parameter, and surgeons should apply traction and retractor positioning with caution when the acromiohumeral distance is reduced.

While studies of Shinagawa et al. and Patel et al. didn’t demonstrate an increased risk of nerve events for patients with smoking history [21, 30], our study identified a significant association (*p* = 0.02). Binomial logistic regression demonstrates an 11-fold increased risk for nerve events in smokers. This may be explained by the compromised tissue oxygenation seen in smokers, potentially resulting in transient nerve ischemia during nerve manipulations and traction. Peripheral nerves are more prone to injury in patients with a smoking history.

The effect of preoperative shoulder ROM on nerve events is controversial in the literature, conflicting results were reported in previous studies. Parisien et al. reported an association between lower degrees of forward flexion and increased nerve events [18], while Nagda et al. found no such relationship in postoperative EMG assessments [17]. In this study, higher degrees of preoperative internal rotation were associated with an increased number of nerve events (*p* = 0.05). This may be caused because of anterior capsular tightness, which could increase traction on neural structures during externally rotated positions such as dislocation and humeral preparation phases. This effect may contribute to nerve injury.

This study has some limitations. Although our cohort exceeded the calculated number required for statistical power, to apply multivariable adjustment analysis for the confounders such as retractor position and extremity position, a larger cohort is still needed to validate the findings. Another limitation is that all surgeries were performed by a single surgeon in a single center. This may limit the generalizability of the results. In addition to that, radiological measurements were performed by a single surgeon. This may cause a measurement error and influence the threshold of 3.7 mm. Also, no patients developed clinically evident postoperative nerve injury. Larger cohorts including cases with clinically evident nerve injury are needed to assess the relationship between intraoperative neuromonitoring findings and post-operative neurological outcomes. Although smoking history is associated with higher risk of nerve events, logistic regression resulted in a wide confidence interval due to relatively small sample size and the number of nerve events. This result should be re-evaluated in larger cohorts.

A major strength of our study is the use of IONM for nerve assessment. With its use, we concluded that nerve events occur notably in glenoid preparation phase and especially the musculocutaneous and radial nerve are at risk of injury. Additionally, this study proposes a novel cutoff value for acromiohumeral distance difference as a predictor of nerve events. While previous studies focused on extremity lengthening, no prior study has considered this radiological parameter for nerve events in RTSA. IONM may help detect transient nerve injury intraoperatively, which may allow timely intraoperative modifications that could reduce the risk of permanent nerve damage.

## Conclusıon

In conclusion, the musculocutaneous and radial nerves were affected more frequently than other nerves during RTSA. Nerve events are more commonly observed during the glenoid preparation phase, likely due to the excessive traction and improper retractor positioning. A preoperative acromiohumeral distance difference of greater than 3.7 mm may serve as a radiographic marker to identify patients at increased risk of nerve injury. A smoking history increased the risk of intraoperative nerve events 11-fold. The use of IONM during RTSA surgery has a potential preventive effect in addition to its diagnostic role.

## Supplementary Information

Supplementary Material 1.

Supplementary Material 2.

## Data Availability

All raw data is available as a supplemental file and uploaded to submission system.

## Abbreviations

AHD: Acromiohumeral distance
CTA: Cuff tear arthropathy
IONM: Intraoperative neuromonitorization
RTSA: Reverse total shoulder arthroplasty
