# Supplementary material for: Evaluation of the effects of extremity elongation on brachial plexus nerves via intraoperative neuromonitoring in patients undergoing reverse total shoulder arthroplasty
Source: BMC Musculoskelet Disord. 2026 Feb 19;27:252. doi: 10.1186/s12891-026-09633-z (PMC13019714; doi:10.1186/s12891-026-09633-z)
Supplement: Supplementary file 1 — Supplementary Material 1. [file 12891_2026_9633_MOESM1_ESM.pdf]

ETİK KURUL BİLGİLERİ	ETİK KURULUN ADI	**İSTANBUL TIP FAKÜLTESİ KLİNİK ARAŞTIRMALARI ETİK KURULU**
	AÇIK ADRESİ:	**İSTANBUL ÜNİVERSİTESİ İSTANBUL TIP FAKÜLTESİ - FATİH/İSTANBUL**
	TELEFON	**0 (212) 414 21 53**
	FAKS	**0 (212) 414 21 53**
	E-POSTA	**itfetikkurul@istanbul.edu.tr.**

BAŞVURU BİLGİLERİ	ARAŞTIRMANIN AÇIK ADI	**"Ters Omuz Protezi Uygulanan Hastalarda Peroperatif Yumuşak Doku Gergınlığı ve Uzamasinin Brakıal Pleksus Sınırlerı Üzerındekı Etkısının Nöromonıtörızasyon ile Değerlendirilmesi"**			
	ARAŞTIRMA PROTOKOL KODU	**---**			
	KOORDİNATÖR/SORUMLU ARAŞTIRMACI UNVANI/ADI/SOYADI	**Prof. Dr. Ali ERŞEN**			
	KOORDİNATÖR/SORUMLU ARAŞTIRMACININ UZMANLIK ALANI	**Ortopedi ve Travmatoloji**			
	KOORDİNATÖR/SORUMLU ARAŞTIRMACININ BULUNDUĞU MERKEZ	**İstanbul Üniversitesi İstanbul Tıp Fakültesi Ortopedi ve Travmatoloji Anabilim Dalı**			
	DESTEKLEYİCİ	**---**			
	DESTEKLEYİCİNİN YASAL TEMSİLCİSİ	**---**			
	ARAŞTIRMANIN FAZI	FAZ 1	☐		
		FAZ 2	☐		
		FAZ 3	☐		
		FAZ 4	☐		
	ARAŞTIRMANIN TÜRÜ	Yeni Bir Endikasyon	☐		
		Yüksek Doz Araştırması	☐		
		Diğer ise belirtiniz :			
	ARAŞTIRMAYA KATILAN MERKEZLER	TEK MERKEZ ■	ÇOK MERKEZLİ ☐	ULUSAL ■	ULUSLAR ARASI ☐

Bu belge 5070 sayılı Elektronik İmza Kanununu Gereğince E-imzalıdır.
Doğrulamak için : https://www.turkiye.gov.tr/istanbul-universitesi-ebys?eD=BSAY6VPLCC&eS=2310947

ARAŞTIRMANIN AÇIK ADI	**"Ters Omuz Protezi Uygulanan Hastalarda Peroperatif Yumuşak Doku Gerginliği ve Uzamasinin Brakial Pleksus Sinirleri Üzerindeki Etkisinin Nöromonitörizasyon ile Değerlendirilmesi"**

	Belge Adı	Tarihi	Versiyon Numarası	Dili
DEĞERLENDİRİLEN BELGELER	ARAŞTIRMA PROTOKOLÜ	■		Türkçe ■ İngilizce □ Diğer □
	BİLGİLENDİRİLMİŞ GÖNÜLLÜ OLUR FORMU	■		Türkçe ■ İngilizce □ Diğer □
	OLGU RAPOR FORMU	□		Türkçe □ İngilizce □ Diğer □
	ARAŞTIRMA BROŞÜRÜ	□		Türkçe □ İngilizce □ Diğer □
DEĞERLENDİRİLEN DİĞER BELGELER	**Belge Adı**		**Açıklama**	
	TÜRKÇE ETİKET ÖRNEĞİ	□		
	SİGORTA	□		
	ARAŞTIRMA BÜTÇESİ	■		
	BİYOLOJİK MATERYEL TRANSFER FORMU	□		
	HASTA KARTI/GÜNLÜKLERİ	□		
	İLAN	□		
	YILLIK BİLDİRİM	□		
	SONUÇ RAPORU	□		
	GÜVENLİLİK BİLDİRİMLERİ	□		
	DİĞER:	■	Anabilim Dalı Başkanlığından Üst Yazı ve Akademik Kurul Kararı, Literatür Kaynağı, Sorumluluk Paylaşım Belgesi, Olgu Rapor Formu, İlgili Elemanların Bilgilendirildiğine Dair Belge, CV	
KARAR BİLGİLERİ	**Karar No: 24**	**Tarih : 01/12/2023**		
	İstanbul Üniversitesi İstanbul Tıp Fakültesi Ortopedi ve Travmatoloji Anabilim Dalında görevli **Prof. Dr. Ali ERŞEN' in** sorumluluğunda ve **Dr. Dağhan KOYUNCU' nun** yürüteceği yukarıda bilgileri verilen araştırma başvuru dosyası ile ilgili belgeler araştırmanın gerekçe, amaç, yaklaşım ve yöntemleri dikkate alınarak incelenmiş, gerçekleştirilmesinde etik ve bilimsel sakınca bulunmadığına toplantıya katılan Etik Kurul üye tam sayısının salt çoğunluğu ile karar verilmiştir.			

**İSTANBUL TIP FAKÜLTESİ KLİNİK ARAŞTIRMALARI ETİK KURULU**	
**ÇALIŞMA ESASI**	**Beşeri Tıbbi Ürünlerin Klinik Araştırmaları Hakkında Yönetmelik, Tıbbi Cihaz Klinik Araştırmaları Yönetmeliği, İyi Klinik Uygulamaları Kılavuzu**
**BAŞKANIN UNVANI / ADI / SOYADI:**	**Prof. Dr. A. Yağız ÜRESİN**

Unvanı/Adı/Soyadı	Uzmanlık Alanı	Kurumu	Cinsiyet		Araştırma ile ilişki *		Katılım **		İmza
**Prof. Dr. A. Yağız ÜRESİN**	**Farmakoloji ve Klinik Farmakoloji**	**İstanbul Tıp Fakültesi (Etik Kurul Başkanı)**	E ■	K □	E □	H ■	E ■	H □	e-imza
**Prof. Dr. Berrin UMMAN**	**Kardiyoloji**	**İstanbul Tıp Fakültesi (Etik Kurul Başkan Yardımcısı)**	E □	K ■	E □	H ■	E ■	H □	e-imza
**Prof. Dr. Ahmet GÜL**	**Romatoloji**	**İstanbul Tıp Fakültesi**	E ■	K □	E □	H ■	E ■	H □	e-imza
**Prof. Dr. Fatma Aytül UYAR**	**Fizyoloji**	**İstanbul Tıp Fakültesi**	E □	K ■	E □	H ■	E ■	H □	e-imza

Yönetmelik kapsamı dışında kalan araştırmalar Etik Kurul bünyesinde oluşturulmuş 4 kişilik alt komisyon tarafından değerlendirilmekte olup Sağlık Bakanlığı iznine tabi değildir.

"Kişisel Verilerin Korunması Kanunu kapsamında çalışmaya katılan gönüllülerin verilerinin korunması ile ilgili tedbirleri almak Araştırmacının sorumluluğundadır"

Bu belge 5070 sayılı Elektronik İmza Kanununu Gereğince E-imzalıdır.
Doğrulamak için : https://www.turkiye.gov.tr/istanbul-universitesi-ebys?eD=BSAY6VPLCC&eS=2310947
